# Bromination of *endo-*7-norbornene derivatives revisited: failure of a computational NMR method in elucidating the configuration of an organic structure

**DOI:** 10.3762/bjoc.19.56

**Published:** 2023-06-02

**Authors:** Demet Demirci Gültekin, Arif Daştan, Yavuz Taşkesenligil, Cavit Kazaz, Yunus Zorlu, Metin Balci

**Affiliations:** 1 Askale Vocational College, Department of Metallurgical Program, Atatürk University 25500 Erzurum, Turkey; 2 Department of Chemistry, Atatürk University, 25240 Erzurum, Turkey; 3 Faculty of Education, Atatürk University, 25240 Erzurum, Turkey; 4 Department of Chemistry, Gebze Technical University, 41400, Gebze, Turkey; 5 Department of Chemistry, Middle East Technical University, 06800 Ankara, Turkey

**Keywords:** bromination, computational NMR, γ-gauche effect, NMR, NOE-Diff experiments

## Abstract

Previously we reported on the bromination of *endo-*7-bromonorbornene at different temperatures yielding mixtures of addition products. The structural elucidations of the formed compounds were achieved by NMR spectroscopy. Particularly, the γ-gauche effect and long-range couplings were instrumental in assigning the stereochemistry of the adducts. However, in a recent paper, Novitskiy and Kutateladze claimed that based on an applied machine learning-augmented DFT method for computational NMR that the structure of the product, (1*R*,2*R*,3*S*,4*S*,7*s*)-2,3,7-tribromobicyclo[2.2.1]heptane was wrong. With the aid of their computational method, they revised a number of published structures, including ours, and assigned our product the structure (1*R*,2*S*,3*R*,*4S*,7*r*)-2,3,7-tribromobicyclo[2.2.1]heptane. To fit their revised structure, they proposed an alternative mechanism featuring a skeletal rearrangement without the intermediacy of a carbocation. Herein, we are not only confirming the structure originally assigned by us through crucial NMR experiments, we also present the ultimate structural proof by means of X-ray crystallography. Moreover, we disprove the mechanism proposed by the aforementioned authors based on sound mechanistic reasoning and point to an oversight by the authors that led them to an erroneous mechanistic pathway.

## Introduction

Nuclear magnetic resonance (NMR) spectroscopy is one of the most important analytical tools used to determine the structure of organic compounds. NMR not only confirms the connectivity of the atoms in the molecule, appropriate 1D and 2D experiments and a variety of other considerations such as coupling constants, their dependence on dihedral angles, as well as through-space interactions (e.g., the nuclear Overhauser effect) help elucidate the correct stereochemistry and configuration in a molecule, in short, it is the single most important spectroscopic tool aside from X-ray crystallography to provide an accurate ensemble-guided view of the structure, even conformational dynamics in a molecule.

Quantum mechanical/nuclear magnetic resonance (NMR) approaches are used for the configurational assignment of organic compounds. The experimental NMR data (^13^C NMR chemical shifts) are compared with those predicted for all possible theoretical stereoisomers. The correct stereochemistry may be obtained by combining the computed and experimental data [1]. Recently, Novitskiy and Kutateladze have developed a machine learning-augmented DFT method for computational NMR, DU8ML, for fast and ‘accurate’ computational approaches [2]. They applied this computational method to a number of previously published organic compounds and claimed to have revised some structures and proposed new mechanisms for those ‘revised structures’ [3]. This paper impelled us to revisit our original work and assess the validity of Novitskiy and Kutateladze’s claim whether our assignment was indeed wrong.

## Results and Discussion

**Scheme 1:**
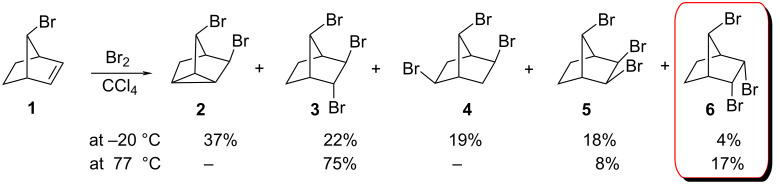
Bromination of *endo-*7-bromonorbornene.

In 2008 we investigated the electrophilic addition of bromine to **1** (7-*endo-*bicyclo[2.2.1]hept-2-ene) at different temperatures and obtained mixtures of the addition products **2–6** (Scheme 1) [4].

**Figure 1:**
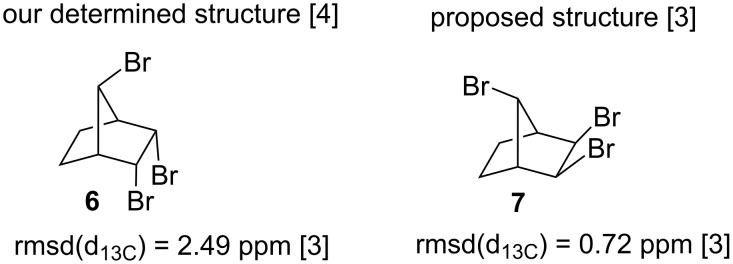
Structure **6** (our assignment) and structure **7** revised by Novitskiy and Kutateladze.

The structures of these compounds **2–6** have been elucidated on the basis of ^1^H and ^13^C NMR spectral data, as well as a number of 2D techniques (APT, HETCOR and COSY), and extensive double resonance experiments. The aforementioned authors, Novitskiy and Kutateladze, however, claimed in their paper [3] that the assigned configuration of product **6** was wrong. They revised the structure **6** to an isomeric symmetric *anti-*7-bis-*exo-*tribromide **7** (Figure 1).

**Figure 2:**
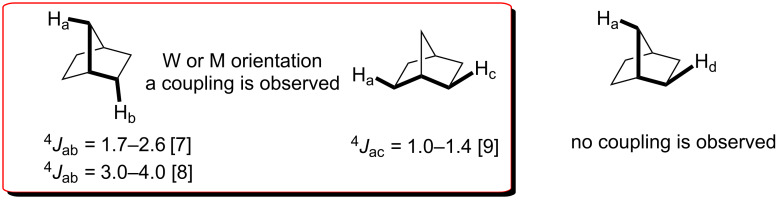
W or M orientaition in norbornane and the corresponding coupling constants.

Below, we will briefly discuss how we initially elucidated the exact configuration of compound **6**. The symmetrical structure of **6** could be characterized easily because of its four-line ^13^C NMR spectrum. However, on the basis of ^13^C NMR data alone we were not able to distinguish between possible symmetrical tribromides. The configurations of the bromine atoms were determined by measuring the couplings between the relevant protons. Proton–proton couplings beyond the three bonds are observed frequently in some strained bicyclic compounds. The long-range coupling exists in a zigzag arrangement (Figure 2). In the case of norbornane if the bonding arrangement of the protons meets the W or M criterion as shown below, long-range couplings between the protons H_a_ and H_b_ as well as between the protons H_a_ and H_c_ are observed [5–6]. However, no coupling is observed between the protons H_a_ and H_d_. These values are extremely important for determining the configuration of the substituents attached to the norbornane skeleton.

**Figure 3:**
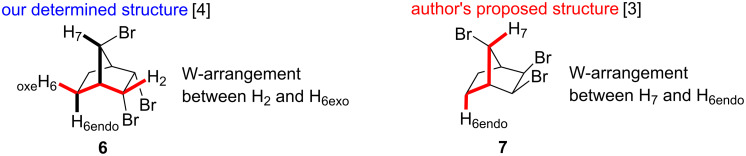
The determined structure **6** by NMR experiments and the proposed structure **7** by computional NMR.

Inspection of both structures **6** and **7** shows that in the case of our structure **6** a long range-coupling should be observed between the protons H_2_ and H_6exo_. However, in the case of the structure **7** proposed by the authors, a long-range coupling between the bridge proton H_7_ and H_6endo_ should be observed (Figure 3) [7–9].

**Figure 4:**
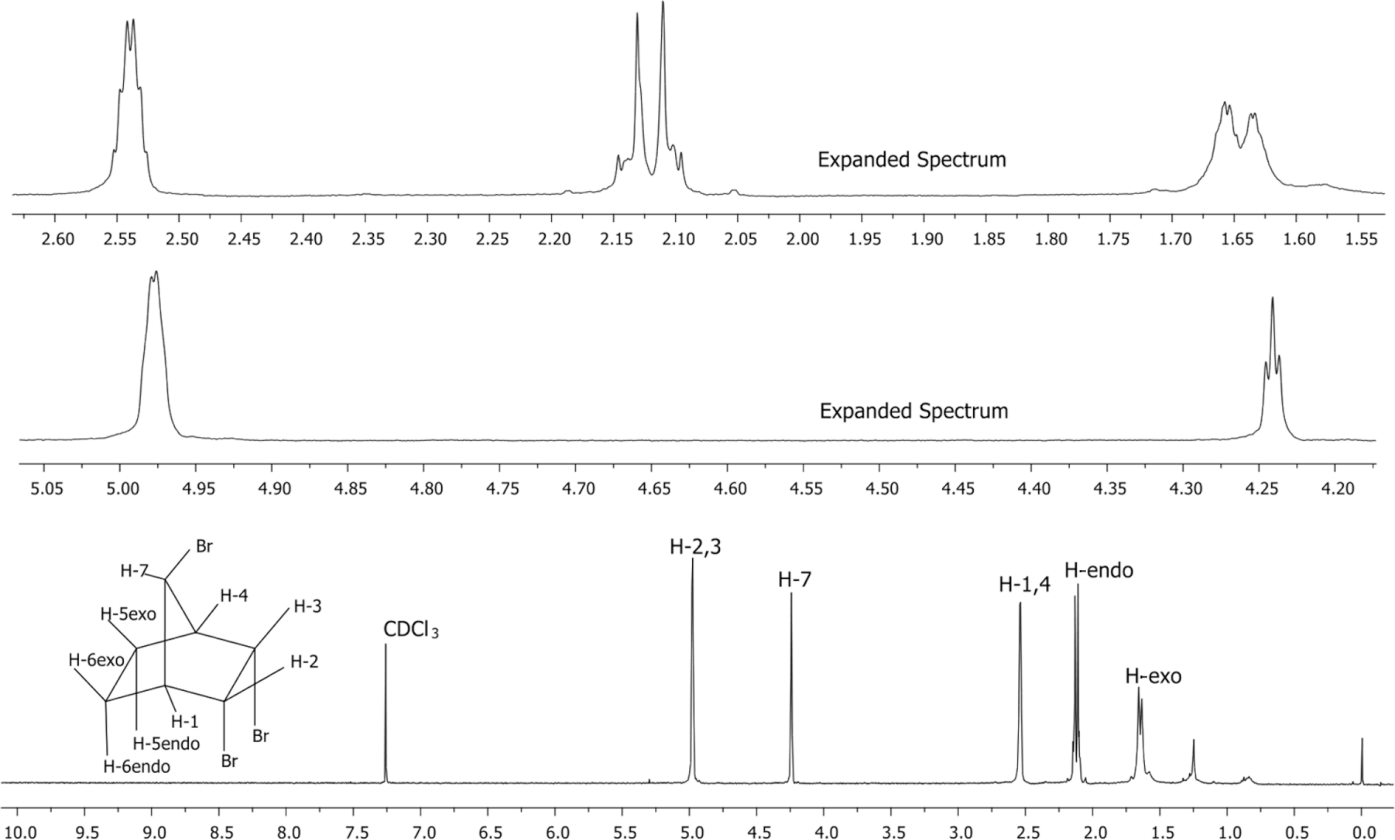
The normal and expanded ^1^H NMR spectra of compound **6**.

The ^1^H NMR spectrum of **6** is in agreement with a symmetrical structure (Figure 1). The bridge proton H_7_ resonates at 4.23 ppm as a triplet that is arising from the vicinal coupling with the bridgehead protons H_1_ and H_4_ (Figure 4).

**Double resonance experiments**: Irradiation at the resonance frequency of the proton H_7_ (4.23 ppm) causes only a change in the resonance signal of the protons H_1_ and H_4_, as expected. This observation clearly shows that there is no proton in **6** with the W arrangement to the proton H_7_. This observation provides evidence that the H_2_ and H_3_ protons have the *exo-*configuration. In other words, the adjacent bromine atoms have the *endo-*configuration (Supporting Information File 1).

Upon irradiation at the resonance frequency (4.97 ppm) of the protons H_2_ and H_3_ no change is observed in the resonance signal of the proton H_7_. This observation proves once again that there is no W arrangement between the H_2_/H_3_ protons and the H_7_ proton, so that the protons H_2_/H_3_ are in the *exo* position and therefore the bromine atoms are in the *endo* position. However, a change in the signals of the H_5exo_ and H_6exo_ protons is observed. This is an expected change since these protons have a zigzag orientation (W orientation, Supporting Information File 1).

Furthermore, irradiation at the resonance frequency of protons H_5exo_ and H_6exo_ does not show any change in the resonance signal of proton H_7_. This observation also proves that there is no W arrangement between the H_5exo_/H_6exo_ protons and the H_7_ proton. The resonance signal of the protons H_2_ and H_3_ collapsed to triplet indicating the zigzag orientation of the relevant protons (Supporting Information File 1).

**Figure 5:**
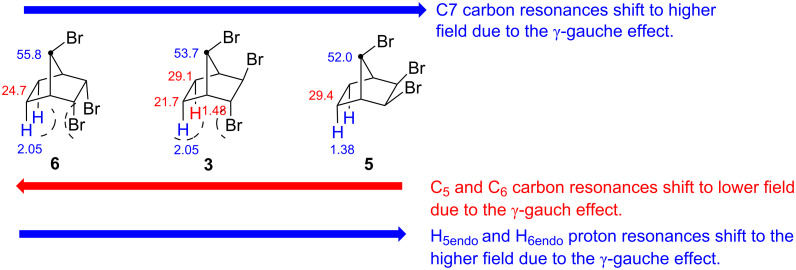
γ-Gauche effects caused by bromine atoms in **3**, **5**, and **6**.

**Structure proof using γ–gauche effect**: The γ-gauche effect is better observed in conformationally rigid systems [10–11]. Since the compounds **3**, **5**, and **6** are also rigid molecules, the γ-gauche effect is also observed in these molecules depending on the configuration of the bromine atoms. The *exo*-orientation of two bromine atoms in **5** causes an upfield shift of the bridge carbon resonance by about 3.8 ppm. Similar effects are also observed in the resonances of the carbons on the ethano bridge as well as the corresponding hydrogen atoms. All these results support the configuration of the bromine atoms in these molecules **3**, **5**, and **6** (Figure 5).

**Figure 6:**
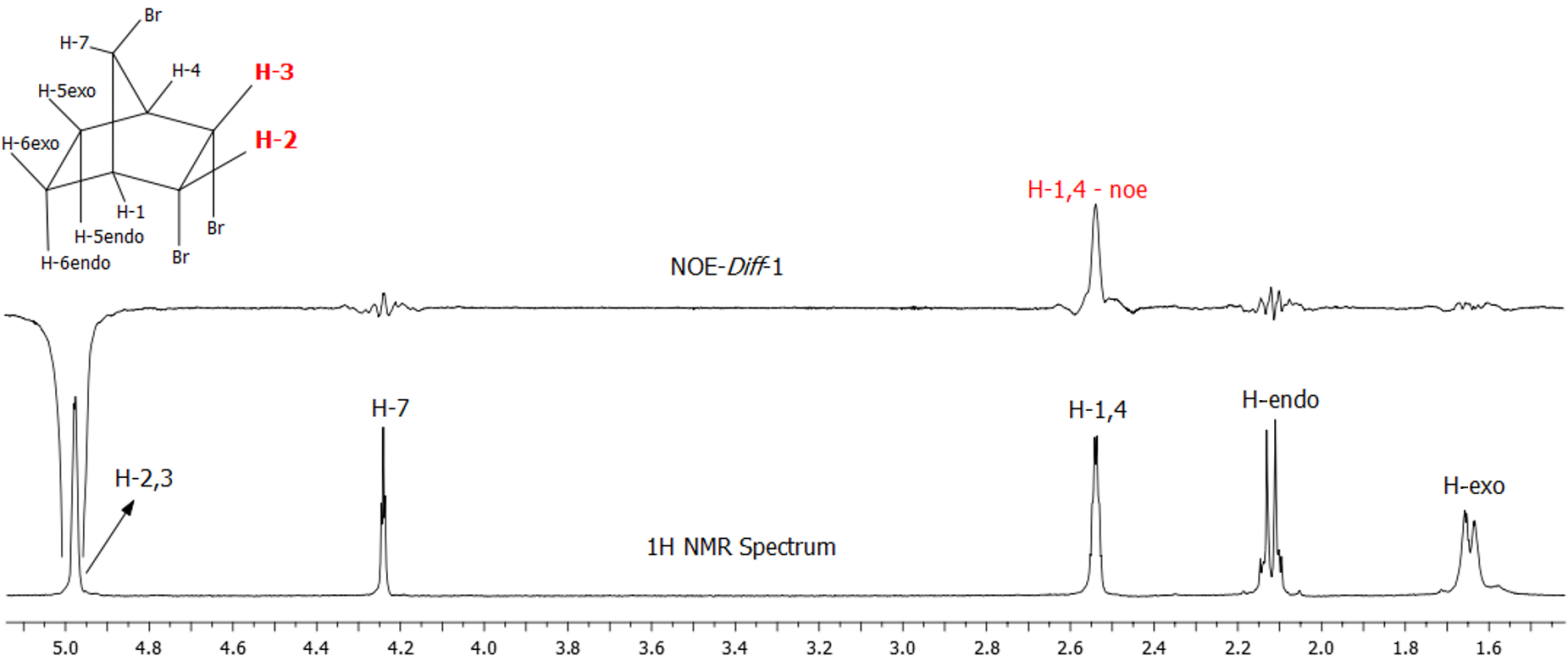
NOE-Diff experiment. Double resonance experiment. Irradiation at the resonance frequency of protons H_2_ and H_3_ (4.97 ppm).

**Structure proof using NOE-Diff experiments:** Irradiation at the resonance frequency of the H_2_ and H_3_ protons produces a positive NOE for the resonance signal of the bridgehead protons H_1_ and H_4_. The fact that only bridgehead protons H_1_ and H_4_ give positive NOE clearly indicates that (i) the configuration of the bromine atom connected to the carbon atom C_7_ is *endo* (located over the CHBr‒CHBr group) and (ii) the H_2_ and H_3_ protons have *exo* configuration (Figure 6).

**Figure 7:**
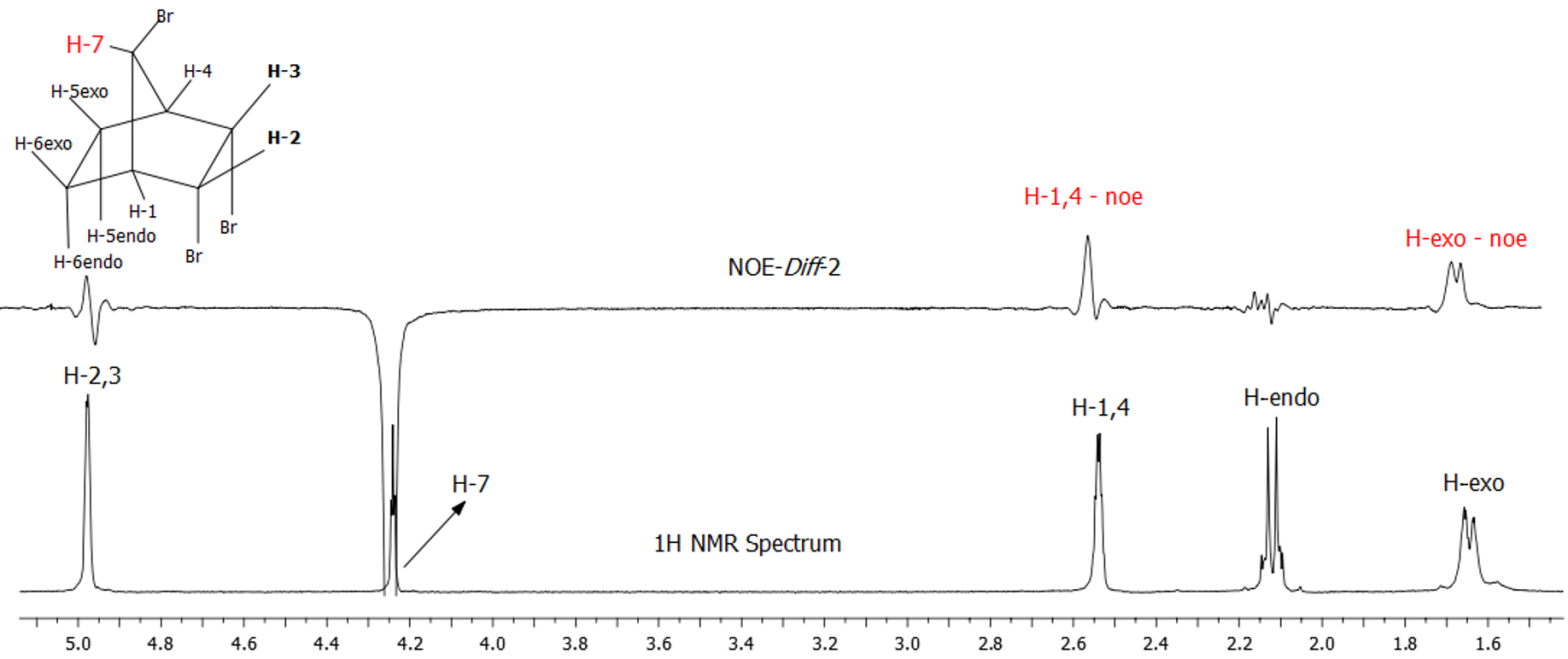
NOE-Diff experiment. Irradiation at the resonance frequency of proton H_7_ (4.23 ppm).

Irradiation at the resonance frequency of the H_7_ proton produces a positive NOE for the resonance signal of the bridgehead protons H_1_ and H_4_ (as expected) as well as for the protons H_5exo_ and H_6exo_. This result clearly indicates that the proton H_7_ is located above the ethano bridge (CH_2_‒CH_2_), in other words, the bromine atom is located over the CHBr‒CHBr linkage (Figure 7). All these results clearly show that the structure **6** is correctly determined by NMR experiments.

**Scheme 2:**
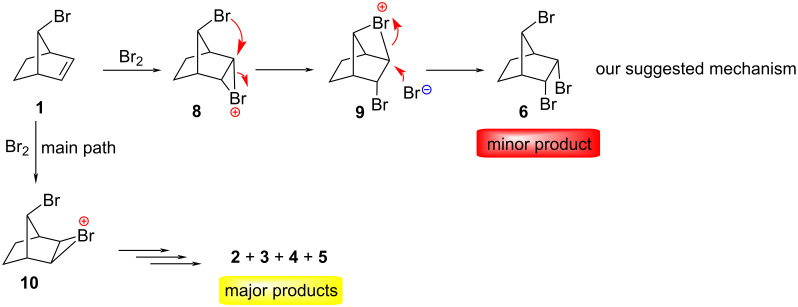
Our mechanism suggested for the formation of **6** [4].

**Mechanism**: For the formation of the compound **6** we proposed the following mechanism: the double bond in norbornene is pyramidalized in the *endo* direction [11]. Norbornene exclusively undergoes an *exo* attack upon treatment with bromine. This *exo* selectivity [12–13] in norbornene, is certainly not surprising since both electronic and steric factors favor attack on the convex face of the pyramidalized double-bond. Electrophilic bromine can attack the double bond in **1** mainly from *exo-*face of the double bond to form the cyclic bromonium ions **10**. The major products, **2**, **3**, **4**, and **5** are formed from the intermediate **10**. Since the bromine atom attached to the C7 carbon atom poses steric hindrance at the *exo* face of the double bond, bromine attacks the double bond also from the *endo* face to form the intermediate **8**. The *endo*-stereochemistry of the bromide attack can be rationalized in terms of neighboring group participation by the bromine atom in the methano bridge (**8**→**9**). Thus, backside attack of the bromine atom on C7 in **8** at C2 of the three-membered bromonium ion can lead to the four-membered bromonium ion **9**. Attack of bromide ion at C-3 of **9** furnishes the *cis*-addition product **6**, which is a minor product (Scheme 2).

**Scheme 3:**
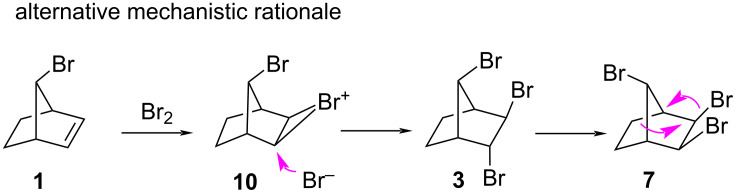
The mechanism suggested by Novitskiy and Kutateladze for the formation of **7** [3].

By contrast, the mechanism proposed by Novitskiy and Kutateladze (Scheme 3) to fit their proposed alternative structure **7** is, in our opinion, not based on sound mechanistic principles: they propose an *exo*-attack (proximal to the Br atom on C7) to form the bromonium ion **10**, which is sterically feasable. However, after the backside attack by the bromide in **10**, the resulting tribromo compound **3** undergoes an unprecedented skeletal rearrangement without a carbocation intermediate to give compound **7**, their proposed alternative structure to **6**. Wagner–Meerwein rearrangements, as postulated by Novitskiy and Kutateladze do not occur spontaneously from neutral compounds without the intermediacy of carbocations. As it turns out, compound **3** in the authors’ scheme is one of the products we described in our original manuscript and is perfectly stable thermally and does not show any tendency to undergo a skeletal rearrangement, in fact with 75% it is the major product at 77 °C [4]. It is somewhat astonishing that the authors have overlooked this fact.

Based on the detailed NMR arguments and experiments we presented above, supported by a sound mechanistic pathway we proposed for the formation of the compound in question **6**, we stand by our original assignment and reject the proposed/’revised’ structure **7** by Novitskiy and Kutateladze.

**Figure 8:**
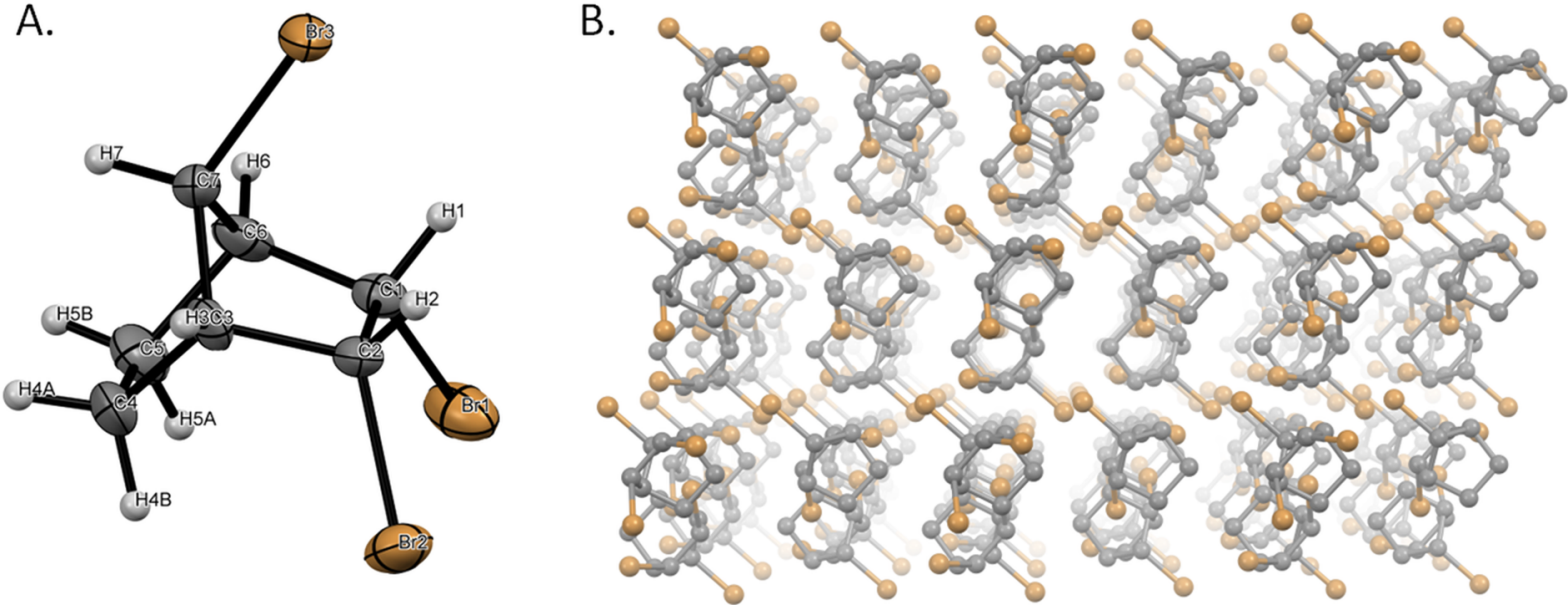
A) Molecular structure of the compound **6** with displacement ellipsoids drawn at the 30% probability level. H-atoms are shown as small spheres of arbitrary radii. B) Perspective view of the crystal packing of compound **6**.

Though we are confident that our assignment is the correct one based on spectroscopic and mechanistic arguments, we decided to settle it irrefutably by X-ray crystallography, which would provide the ultimate structural confirmation. Toward that end, we resynthesized the molecule **6**, isolated a sample and subjected it to single crystal X-ray analysis, which provided the ultimate final piece of evidence (Figure 8).

Additional crystallographic data with CCDC reference number 2201943 has been deposited within the Cambridge Crystallographic Data Center via https://www.ccdc.cam.ac.uk/deposit.

## Conclusion

In conclusion, we have successfully refuted the report by Novitskiy and Kutateladze by carefully analyzing and interpreting the *experimental* NMR data of our structures and confirmed our published structure, and the X-ray structure provided the final piece of evidence in the process.

As a side note, we would like to remind scientists that theory is not the ultimate means for structural elucidations and cannot be used to refute experimental evidence since it has its obvious limitations. However, when used as a complementary tool to experimental work, theory can be very valuable. Relying solely on a computational method in structural assignments and trying to revise published work by others can sometimes result in situations where the theorist may need to re-examine his/her computational approach and try to use it rather in a supportive role in organic chemistry.

## Supporting Information

File 1 Double resonance spectra and X-ray crystal structure.
