# Supplementary material for: Bromination of endo-7-norbornene derivatives revisited: failure of a computational NMR method in elucidating the configuration of an organic structure
Source: Beilstein J Org Chem. 2023 Jun 2;19:764–70. doi: 10.3762/bjoc.19.56 (PMC10241099; doi:10.3762/bjoc.19.56)
Supplement: File 1 — Double resonance spectra and X-ray crystal structure. [file Beilstein_J_Org_Chem-19-764-s001.pdf]

BEILSTEIN JOURNAL OF ORGANIC CHEMISTRY

Supporting Information

for

Bromination of *endo*-7-norbornene derivatives revisited: failure of a computational NMR method in elucidating the configuration of an organic structure

Demet Demirci Gültekin, Arif Daştan, Yavuz Taşkesenligil, Cavit Kazaz, Yunus Zorlu and Metin Balci

Beilstein J. Org. Chem. **2023,** *19,* 764–770. doi:10.3762/bjoc.19.56

Double resonance spectra and X-ray crystal structure

License and Terms: This is a supporting information file under the terms of the Creative Commons Attribution License (https://creativecommons.org/licenses/by/4.0). Please note that the reuse, redistribution and reproduction in particular requires that the author(s) and source are credited and that individual graphics may be subject to special legal provisions.
The license is subject to the *Beilstein Journal of Organic Chemistry* terms and conditions: (https://www.beilstein-journals.org/bjoc/terms)

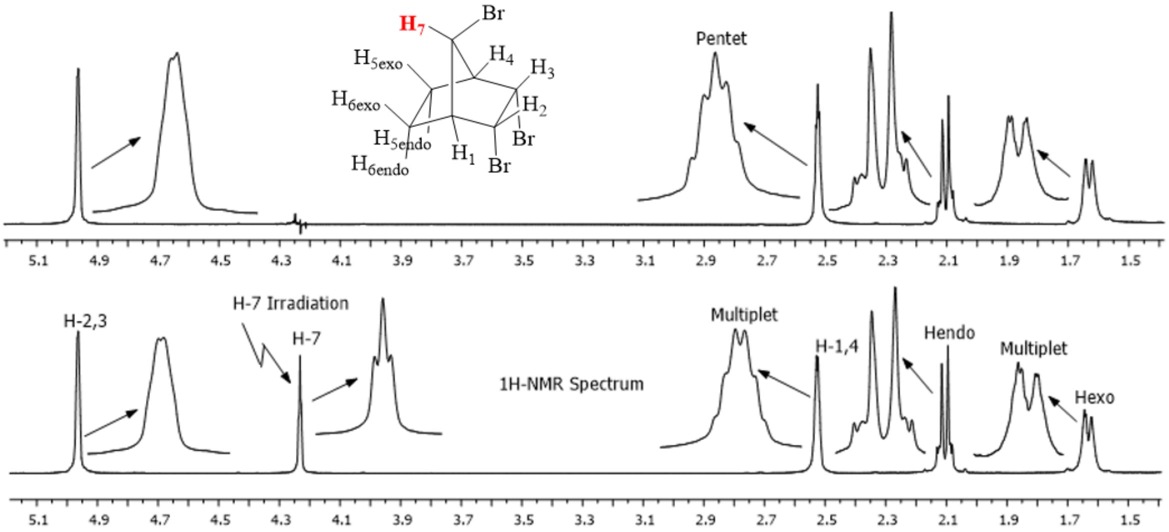

Figure 1. Double resonance experiment. Irradiation at the resonance frequency of proton H_7 (4.23 ppm).

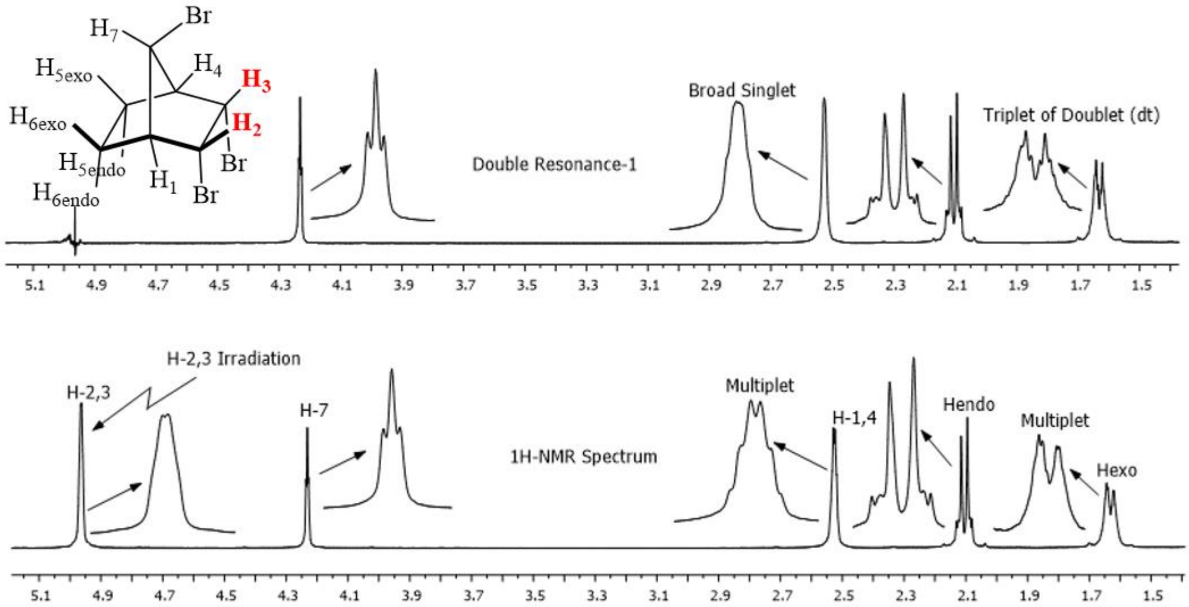

Figure 2. Double resonance experiment. Irradiation at the resonance frequency of protons H_2 and H_3 (4.97 ppm).

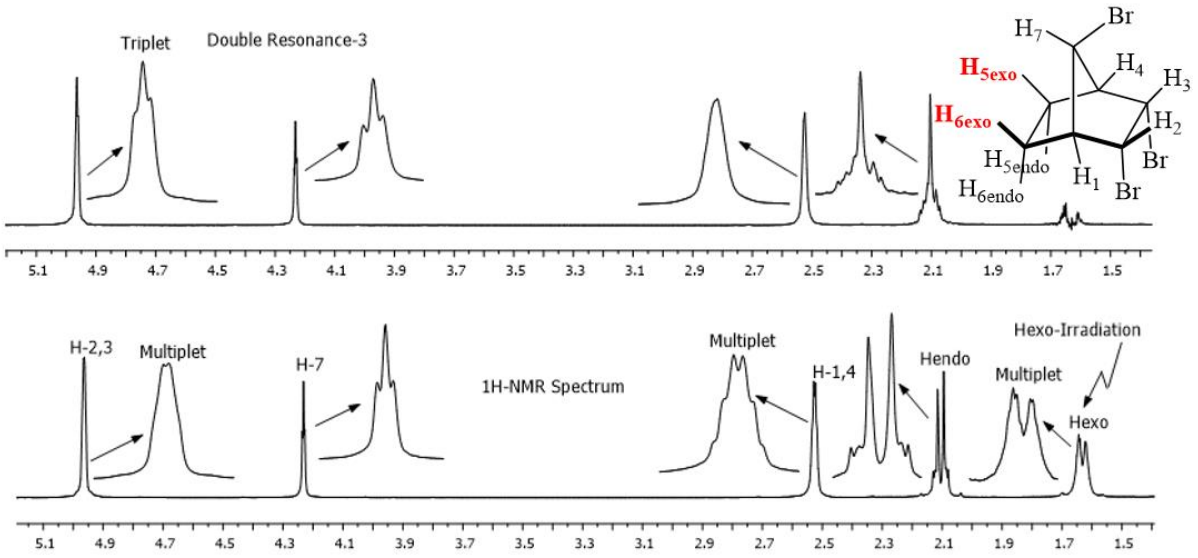

Figure 3. Double resonance experiment. Irradiation at the resonance frequency of protons H_{5exo} and H_{6exo} (1.66 ppm).

X-ray crystallography: Single crystal X-ray diffraction data of **6** were performed on a Bruker APEX II QUAZAR three-circle diffractometer using using monochromatized Mo-K$_{\alpha}$ X-radiation (λ = 0.71073 Å). Indexing, data collection, data reduction[1] and absorption correction[2] were carried out using APEX2.[3] Crystal structure was solved using SHELXT[4] and then refined by full-matrix least-squares refinements on F^2 using the SHELXL[4] in Olex2 Software Package.[5] The aliphatic C-bound H atoms were positioned geometrically and refined using a riding mode. Crystal structure validations, geometrical calculations and drawings were performed using Platon[6] and Mercury software.[7] Bond distances, angles and torsion angles of compound **6** were indicated in **Table S2–S5**. Perspective views of compounds showing the atom-numbering scheme and crystal packing were shown in **Figure S1.** Crystal data and structure refinement details for crystal structure were given in **Table S1**. Additional crystallographic data with CCDC reference number 2201943 has been deposited within the Cambridge Crystallographic Data Center *via* www.ccdc.cam.ac.uk/deposit.

Table S1. Crystal data and structure refinement details for compound **6**.

**CCDC**	2201943
**Empirical formula**	$C_7H_9Br_3$
**Formula weight**	332.87
**Temperature/K**	298
**Crystal system**	Monoclinic
**Space group**	$P2_1/c$
**a/Å**	6.307(2)
**b/Å**	13.8207(10)
**c/Å**	10.4280(10)
**α/°**	90
**β/°**	99.58(2)
**γ/°**	90
**Volume/Å^3**	896.3(3)
**Z**	4
**ρ_{calc}g/cm^3**	2.467
**μ/mm^{-1}**	13.426
**F(000)**	624.0
**Crystal size/mm^3**	0.235 × 0.147 × 0.106
**Radiation**	MoKα (λ = 0.71073)
**2Θ range for data collection/°**	5.896 to 50.054
**Index ranges**	$-7 \leq h \leq 7$, $-16 \leq k \leq 16$, $-12 \leq l \leq 11$
**Reflections collected**	6663
**Independent reflections**	1550 [R_{int} = 0.1010, R_{sigma} = 0.0753]
**Data/restraints/parameters**	1550/0/91
**Goodness-of-fit on F^2**	1.038
**Final R indexes [I>=2σ (I)]**	R_1 = 0.0595, wR_2 = 0.1646
**Final R indexes [all data]**	R_1 = 0.0890, wR_2 = 0.1942
**Largest diff. peak/hole / e Å^{-3}**	1.11/-1.39

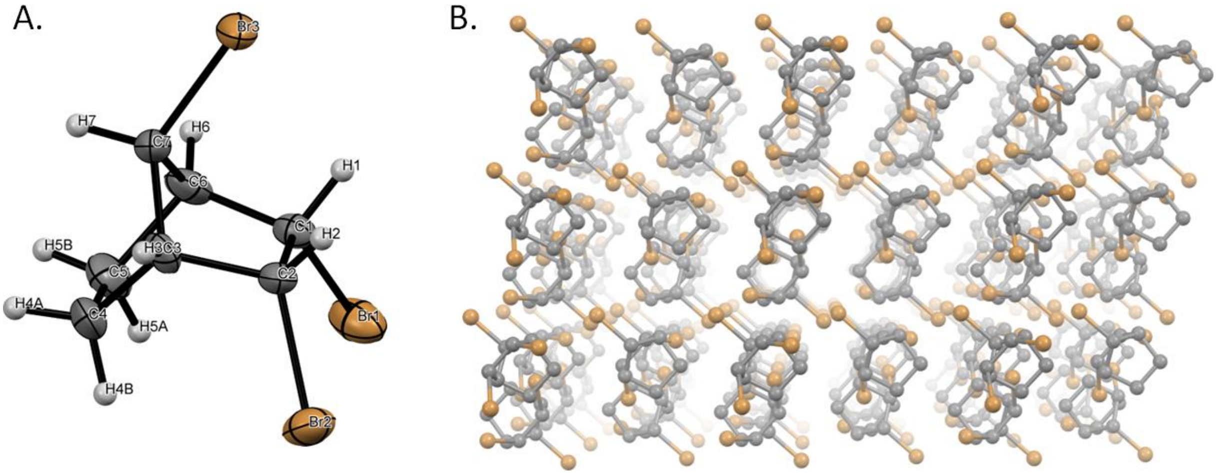

Figure S1. A) Molecular structure of the compound **6** with displacement ellipsoids drawn at the 30% probability level. H-atoms are shown as small spheres of arbitrary radii. B) Perspective view of the crystal packing of compound **6**

Table S2. Bond lengths for compound **6**.

Atom	Atom	Length/Å	Atom	Atom	Length/Å
Br1	C1	1.934(8)	C3	C4	1.532(13)
Br2	C2	1.919(8)	C3	C7	1.509(12)
Br3	C7	1.957(9)	C4	C5	1.546(17)
C1	C2	1.535(13)	C5	C6	1.539(12)
C1	C6	1.491(14)	C6	C7	1.527(15)
C2	C3	1.505(13)			

Table S3. Bond angles for compound **6**.

Atom	Atom	Atom	Angle/°	Atom	Atom	Atom	Angle/°
C2	C1	Br1	117.9(6)	C3	C4	C5	104.4(8)
C6	C1	Br1	114.3(6)	C6	C5	C4	101.5(8)
C6	C1	C2	104.5(7)	C1	C6	C5	110.4(8)
C1	C2	Br2	116.5(6)	C1	C6	C7	99.6(8)
C3	C2	Br2	112.5(6)	C7	C6	C5	101.8(9)
C3	C2	C1	102.6(7)	C3	C7	Br3	115.5(7)
C2	C3	C4	111.5(8)	C3	C7	C6	94.9(7)
C2	C3	C7	100.8(7)	C6	C7	Br3	116.2(6)
C7	C3	C4	99.6(9)				

Table S4. Torsion angles for compound **6**.

A	B	C	D	Angle/°	A	B	C	D	Angle/°
Br1	C1	C2	Br2	-5.9(9)	C2	C3	C7	Br3	-65.4(9)
Br1	C1	C2	C3	-129.2(7)	C2	C3	C7	C6	56.8(8)
Br1	C1	C6	C5	59.9(10)	C3	C4	C5	C6	-3.2(12)
Br1	C1	C6	C7	166.5(5)	C4	C3	C7	Br3	-179.6(7)
Br2	C2	C3	C4	-56.2(10)	C4	C3	C7	C6	-57.5(8)
Br2	C2	C3	C7	-161.0(7)	C4	C5	C6	C1	72.2(11)
C1	C2	C3	C4	69.8(9)	C4	C5	C6	C7	-32.8(11)
C1	C2	C3	C7	-35.1(8)	C5	C6	C7	Br3	178.1(7)
C1	C6	C7	Br3	64.7(8)	C5	C6	C7	C3	56.5(9)
C1	C6	C7	C3	-56.9(8)	C6	C1	C2	Br2	122.3(7)
C2	C1	C6	C5	-70.4(10)	C6	C1	C2	C3	-1.0(8)
C2	C1	C6	C7	36.1(8)	C7	C3	C4	C5	38.4(10)
C2	C3	C4	C5	-67.2(11)					

References

1. SAINT, version 8.34A, Bruker (2013), Bruker AXS Inc., Madison, WI
2. SADABS, version 2014/5, Bruker (2014), Bruker AXS Inc., Madison, WI
3. APEX2, version 2014.11-0, Bruker (2014), Bruker AXS Inc., Madison, WI
4. Sheldrick, G. M. Crystal Structure Refinement with SHELXL. *Acta Crystallogr. Sect. C Struct. Chem*. **2015**, *71*, 3-8.
5. Dolomanov, O. L.; Bourhis, J.; Gildea, R. J.; Howard, J. A. K.; Puschmann, H. *J. Appl. Cryst*. **2009**, *42*, 339-341.
6. Spek, A. L. Structure Validation in Chemical Crystallography. *Acta Crystallogr. Sect. D Biol. Crystallogr*. **2009**, *65*, 148-155.
7. Macrae, C. F.; Sovago, I.; Cottrell, S. J.; Galek, P. T. A.; McCabe, P.; Pidcock, E.; Platings, M.; Shields, G. P.; Stevens, J. S.; Towler, M.; Wood, P. A. *J. Appl. Cryst*. **2020**, 53.
